# Supplementary material for: Systematic temporal analysis of peripheral blood transcriptomes using TrendCatcher identifies early and persistent neutrophil activation as a hallmark of severe COVID-19
Source: bioRxiv. 2021 Dec 7:2021.05.04.442617. Preprint. [Version 3] doi: 10.1101/2021.05.04.442617 (PMC8629189; doi:10.1101/2021.05.04.442617)
Supplement: 1 [file NIHPP2021.05.04.442617V3-supplement-1.pdf]

bioRxiv preprint doi: https://doi.org/10.1101/2021.05.04.442617; this version posted December 7, 2021. The copyright holder for this preprint (which was not certified by peer review) is the author/funder, who has granted bioRxiv a license to display the preprint in perpetuity. It is made available under aCC-BY 4.0 International license.

Innate immune cells					
NK		Monocyte		DC	
Moderate	Severe	Moderate	Severe	Moderate	Severe
708	1,179	1,479	1,845	22	17

Supplement Table 1. Number of DDEGs for each innate immune cell type identified by *TrendCatcher*. Using FDR less than 0.05.

Adaptive immune cells							
B		$CD4^+$ T		$CD8^+$ T		MAIT	
Moderate	Severe	Moderate	Severe	Moderate	Severe	Moderate	Severe
969	1,344	190	1,060	634	817	71	13

Supplement Table 2. Number of DDEGs for each adaptive immune cell type identified by *TrendCatcher*. Using FDR less than 0.05.

bioRxiv preprint doi: https://doi.org/10.1101/2021.05.04.442617; this version posted December 7, 2021. The copyright holder for this preprint (which was not certified by peer review) is the author/funder, who has granted bioRxiv a license to display the preprint in perpetuity. It is made available under aCC-BY 4.0 International license.

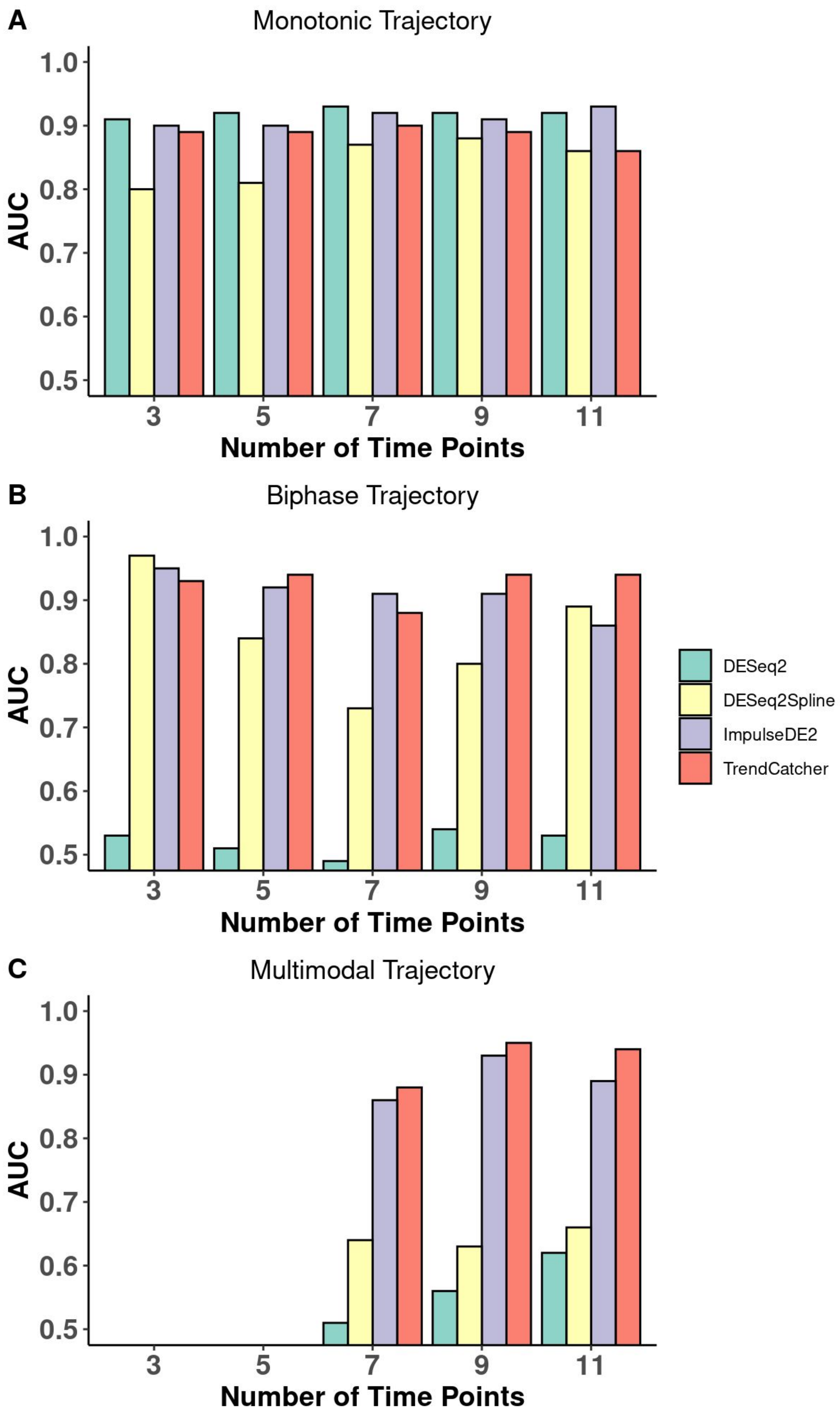

Supplementary Figure 1, related to Figure 1. TrendCatcher prediction performance on different types of trajectories. (A) Prediction performance of *TrendCatcher* across varying numbers of time points for monotonic trajectories. (B) Prediction performance of *TrendCatcher* across varying numbers of time points for biphasic trajectories. (C) Prediction performance of *TrendCatcher* across varying numbers of time points for multimodal trajectories. DESeq2 is shown in green, DESeq2Spline is shown in yellow, ImpulseDE2 is shown in purple, *TrendCatcher i*s shown in red.

bioRxiv preprint doi: https://doi.org/10.1101/2021.05.04.442617; this version posted December 7, 2021. The copyright holder for this preprint (which was not certified by peer review) is the author/funder, who has granted bioRxiv a license to display the preprint in perpetuity. It is made available under aCC-BY 4.0 International license.

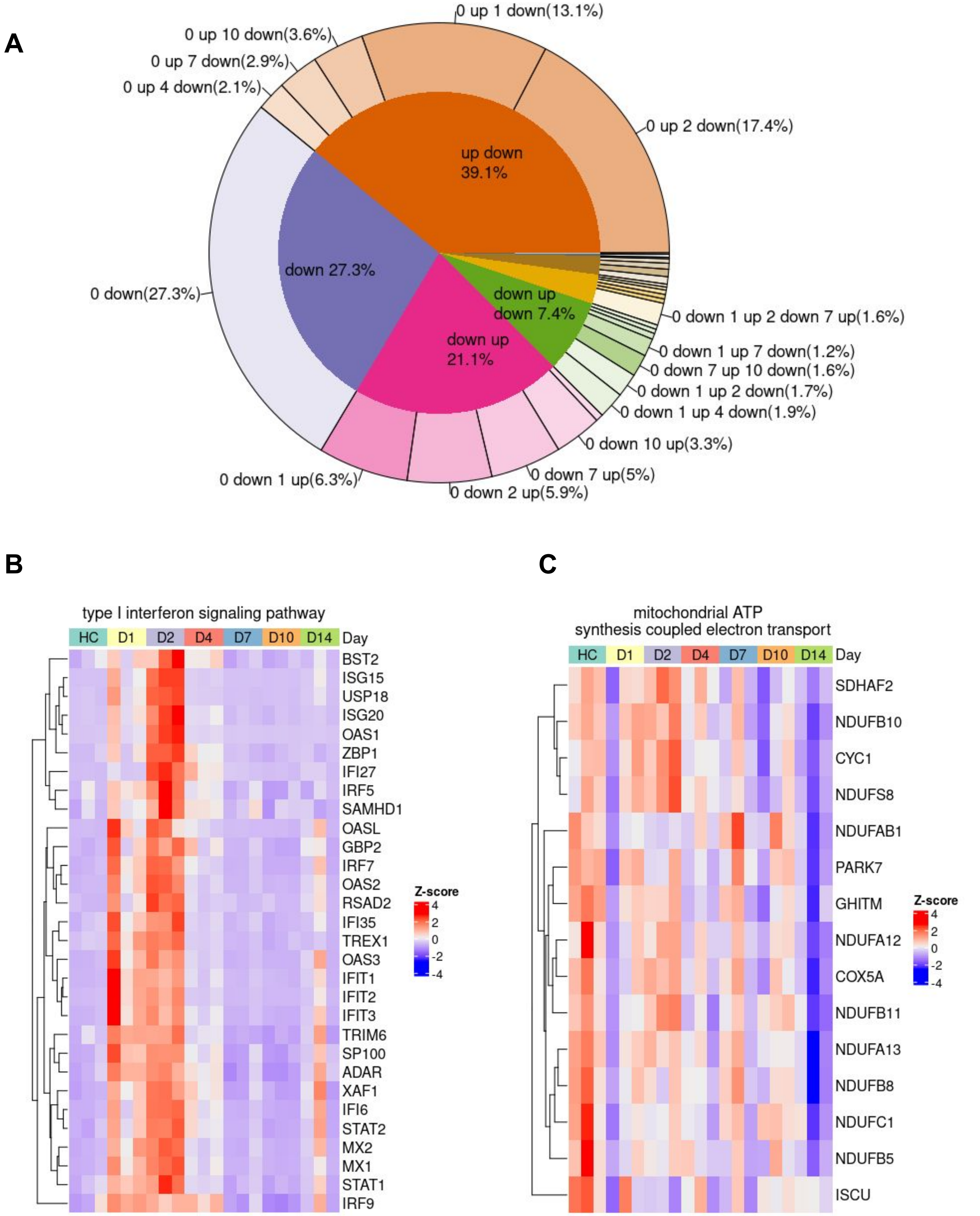

Supplementary Figure 2, related to Figure 2. Trajectory pattern composition of DDEGS and dynamic gene signatures from highlighted pathways. (A) Hierarchical pie chart shows the composition of trajectory patterns of DDEGs identified in a non-human primate bulk peripheral blood mRNA (Aid et al.). (B) Dynamic gene signatures for SARS-CoV-2 infection from type I interferon signaling pathway. (C) Dynamic gene signatures response for SARS-CoV-2 infection from mitochondrial ATP synthesis coupled electron transport pathway. Color represents normalized z-score for gene expression.

bioRxiv preprint doi: https://doi.org/10.1101/2021.05.04.442617; this version posted December 7, 2021. The copyright holder for this preprint (which was not certified by peer review) is the author/funder, who has granted bioRxiv a license to display the preprint in perpetuity. It is made available under aCC-BY 4.0 International license.

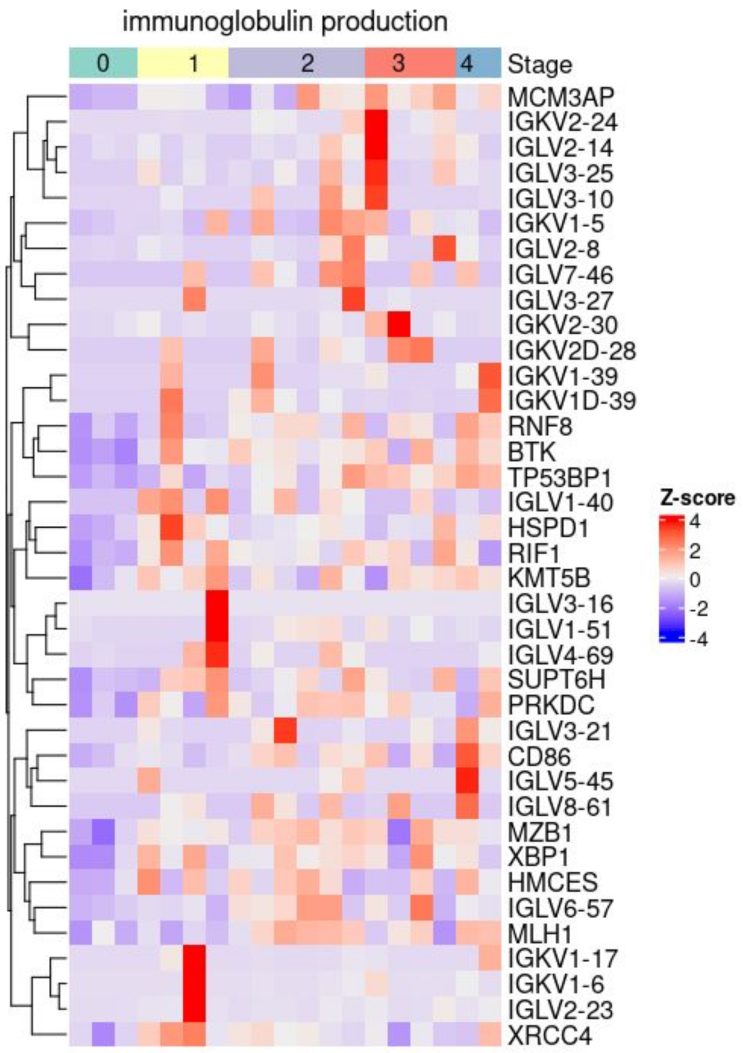

Supplementary Figure 3, related to Figure 3. Dynamic gene signatures of immunoglobulin pathway. Heatmap showing B cell's 38 DDEGs identified from immunoglobulin production process. Color represents normalized z-score of gene expression.

bioRxiv preprint doi: https://doi.org/10.1101/2021.05.04.442617; this version posted December 7, 2021. The copyright holder for this preprint (which was not certified by peer review) is the author/funder, who has granted bioRxiv a license to display the preprint in perpetuity. It is made available under aCC-BY 4.0 International license.

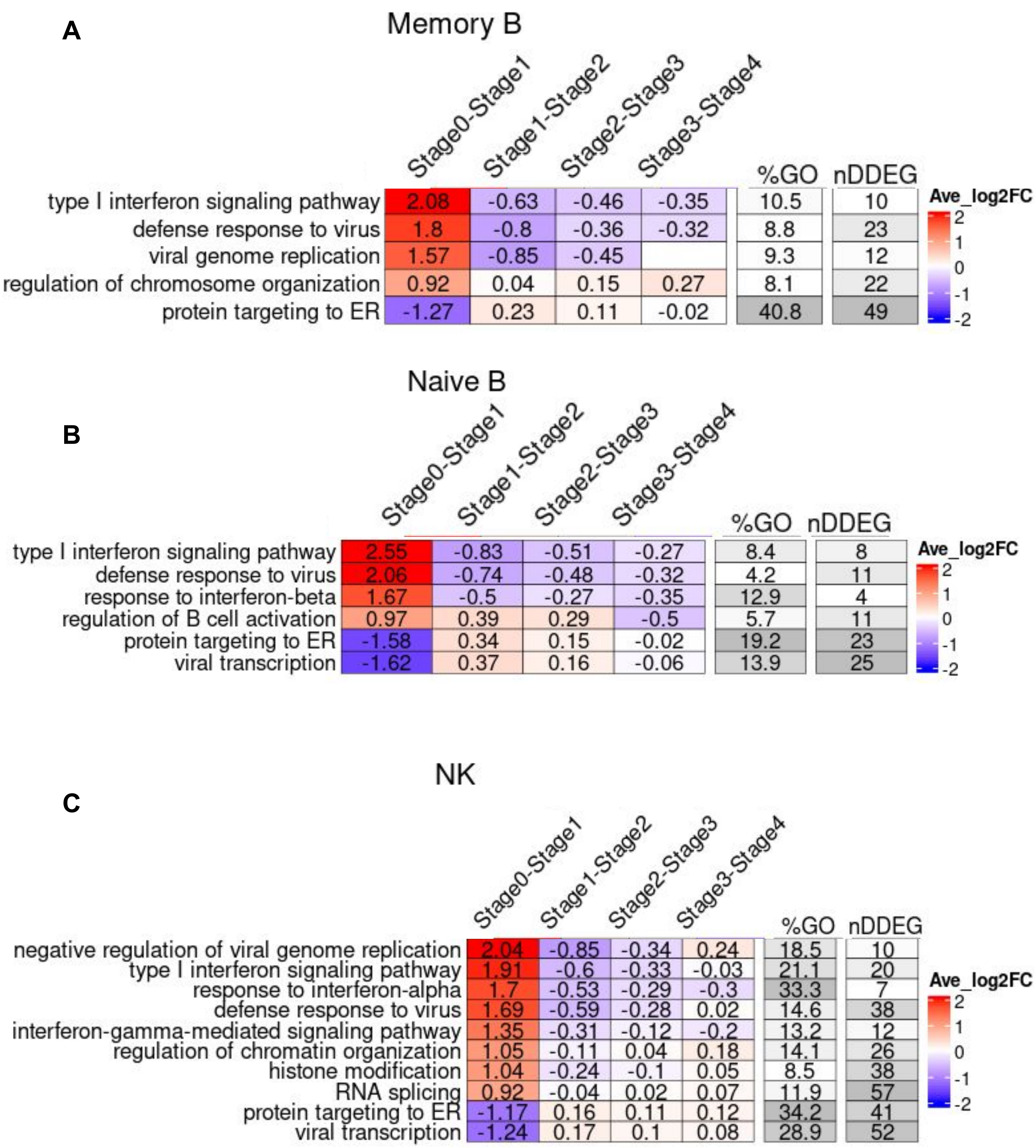

Supplementary Figure 4, related to Figure 3. TimeHeatmap of B cells and NK cells. (A) *TimeHeatmap* of Memory B cells. (B) *TimeHeatmap* of Naive B cells. (C) *TimeHeatmap* of NK cells.

bioRxiv preprint doi: https://doi.org/10.1101/2021.05.04.442617; this version posted December 7, 2021. The copyright holder for this preprint (which was not certified by peer review) is the author/funder, who has granted bioRxiv a license to display the preprint in perpetuity. It is made available under aCC-BY 4.0 International license.

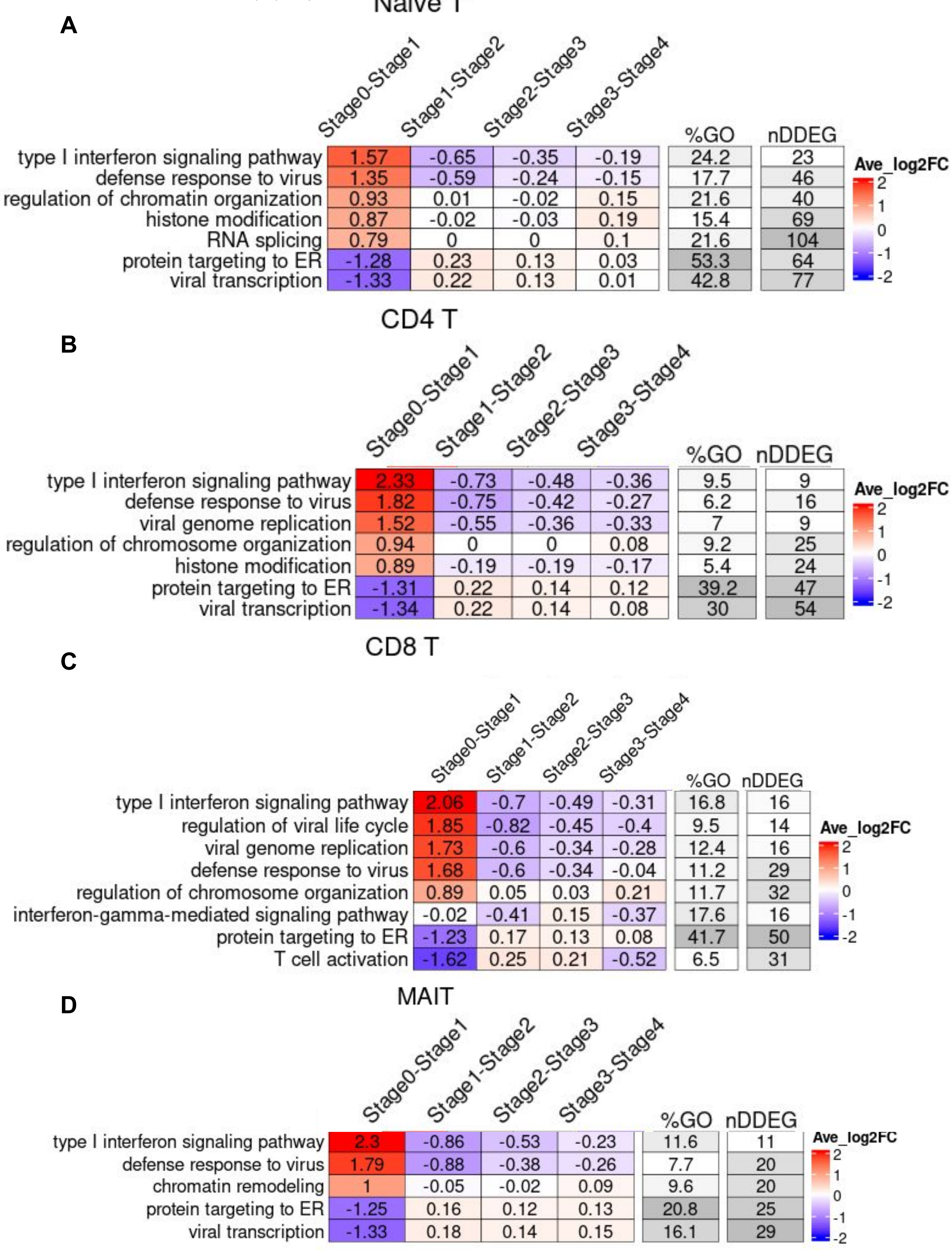

Supplementary Figure 5, related to Figure 3. TimeHeatmap of T cells. (A) *TimeHeatmap* of Naive T cells. (B) *TimeHeatmap* of CD4$^+$ T cells. (C) *TimeHeatmap* of CD8$^+$ T cells. (D) *TimeHeatmap* of MAIT cells.

bioRxiv preprint doi: https://doi.org/10.1101/2021.05.04.442617; this version posted December 7, 2021. The copyright holder for this preprint (which was not certified by peer review) is the author/funder, who has granted bioRxiv a license to display the preprint in perpetuity. It is made available under aCC-BY 4.0 International license.

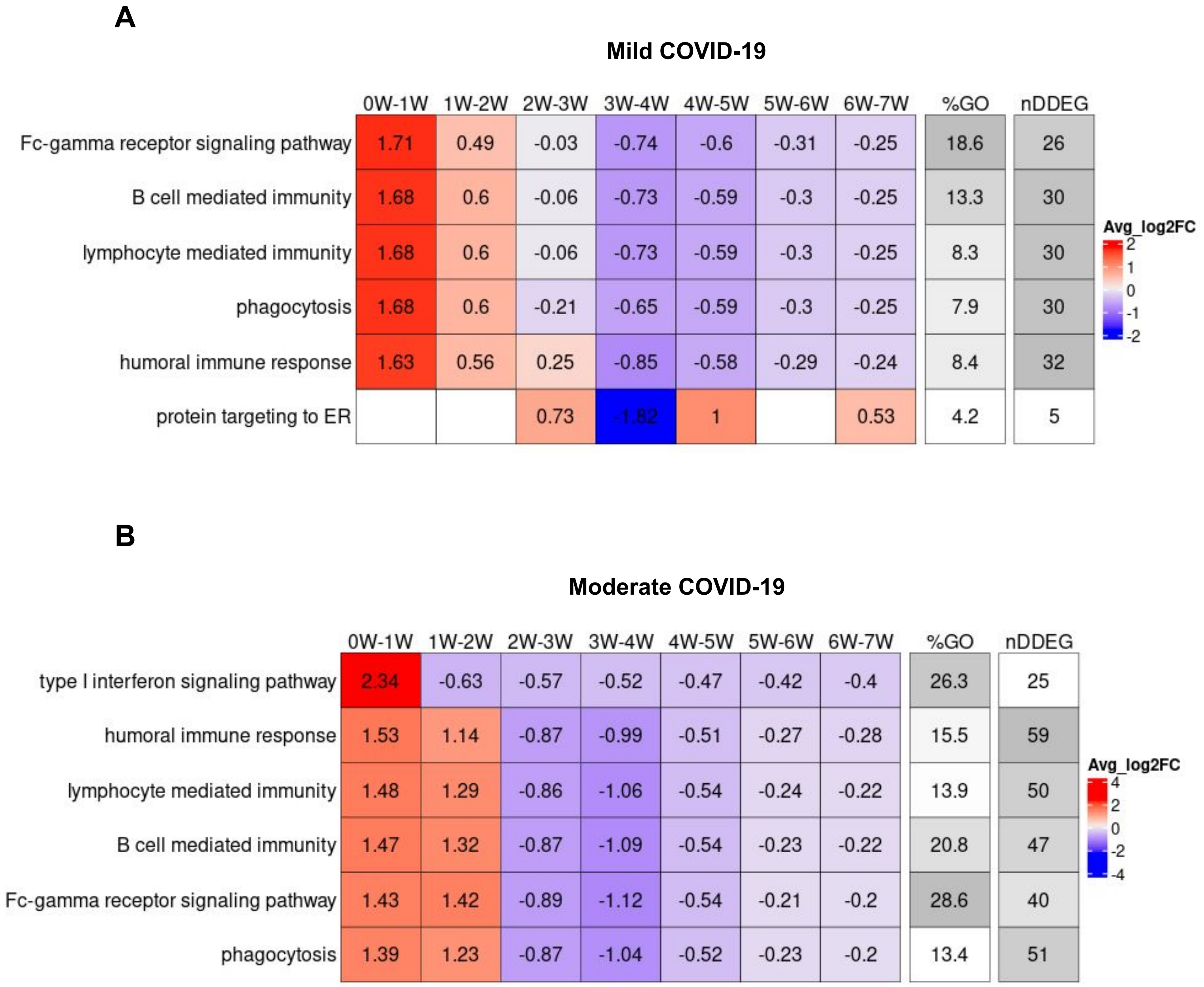

Supplementary Figure 6, related to Figure 4. TimeHeatmap of mild and moderate COVID-19. (A) *TimeHeatmap* of top dynamic GO terms found in severe COVID-19 shown in mild group. (B) *TimeHeatmap* of top dynamic GO terms found in severe COVID-19 shown in moderate group.

bioRxiv preprint doi: https://doi.org/10.1101/2021.05.04.442617; this version posted December 7, 2021. The copyright holder for this preprint (which was not certified by peer review) is the author/funder, who has granted bioRxiv a license to display the preprint in perpetuity. It is made available under aCC-BY 4.0 International license.

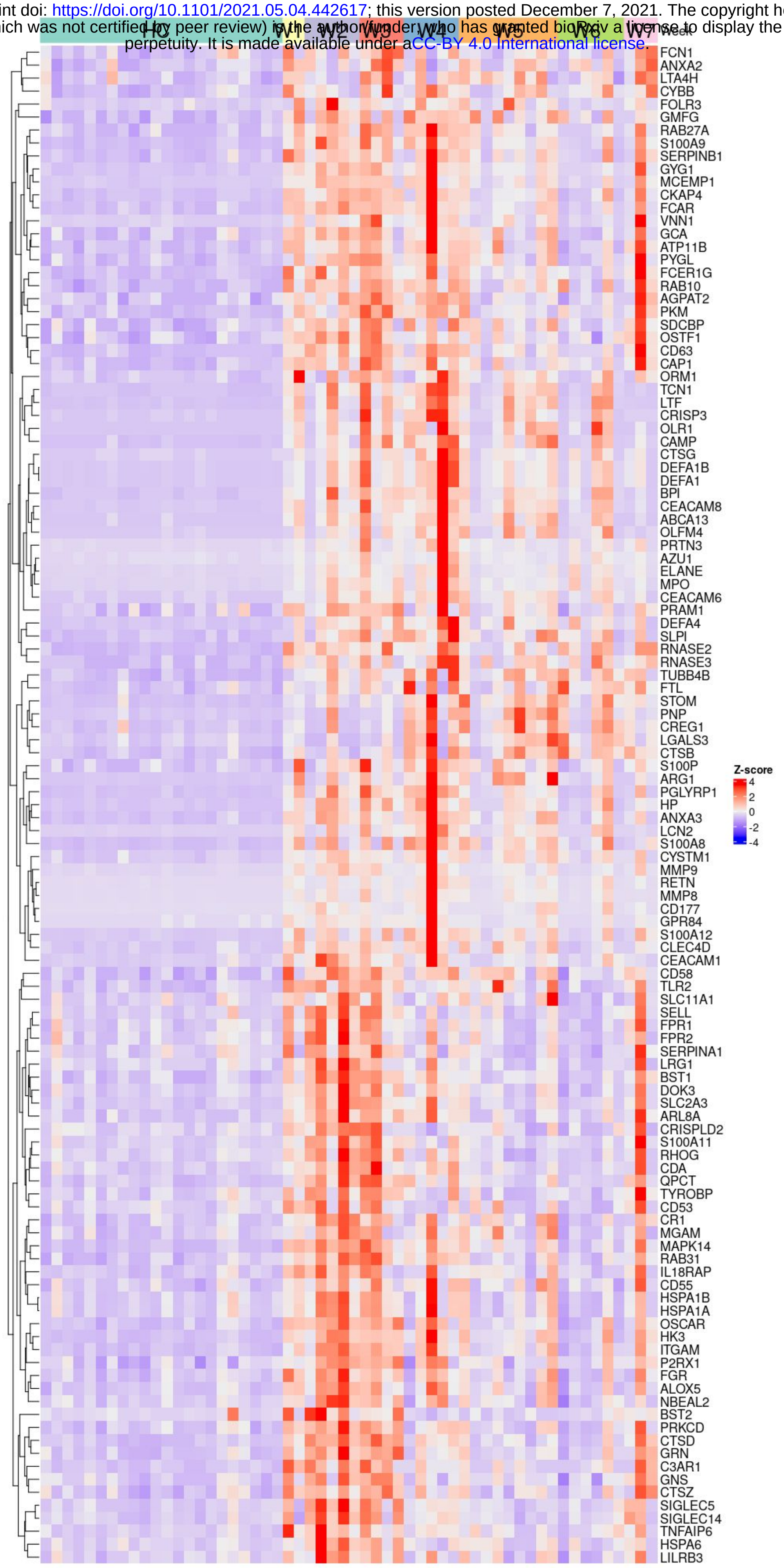

Supplementary Figure 7, related to Figure 4. Dynamic gene signatures of neutrophil activation from severe COVID-19. Heatmap of severe COVID-19 DDEGs identified from the neutrophil activation pathway. The heatmap is ordered by week following infection with healthy controls (HC) on the left. Color represents Z-score normalized expression values. Each column represents one sample.

bioRxiv preprint doi: https://doi.org/10.1101/2021.05.04.442617; this version posted December 7, 2021. The copyright holder for this preprint (which was not certified by peer review) is the author/funder, who has granted bioRxiv a license to display the preprint in perpetuity. It is made available under aCC-BY 4.0 International license.

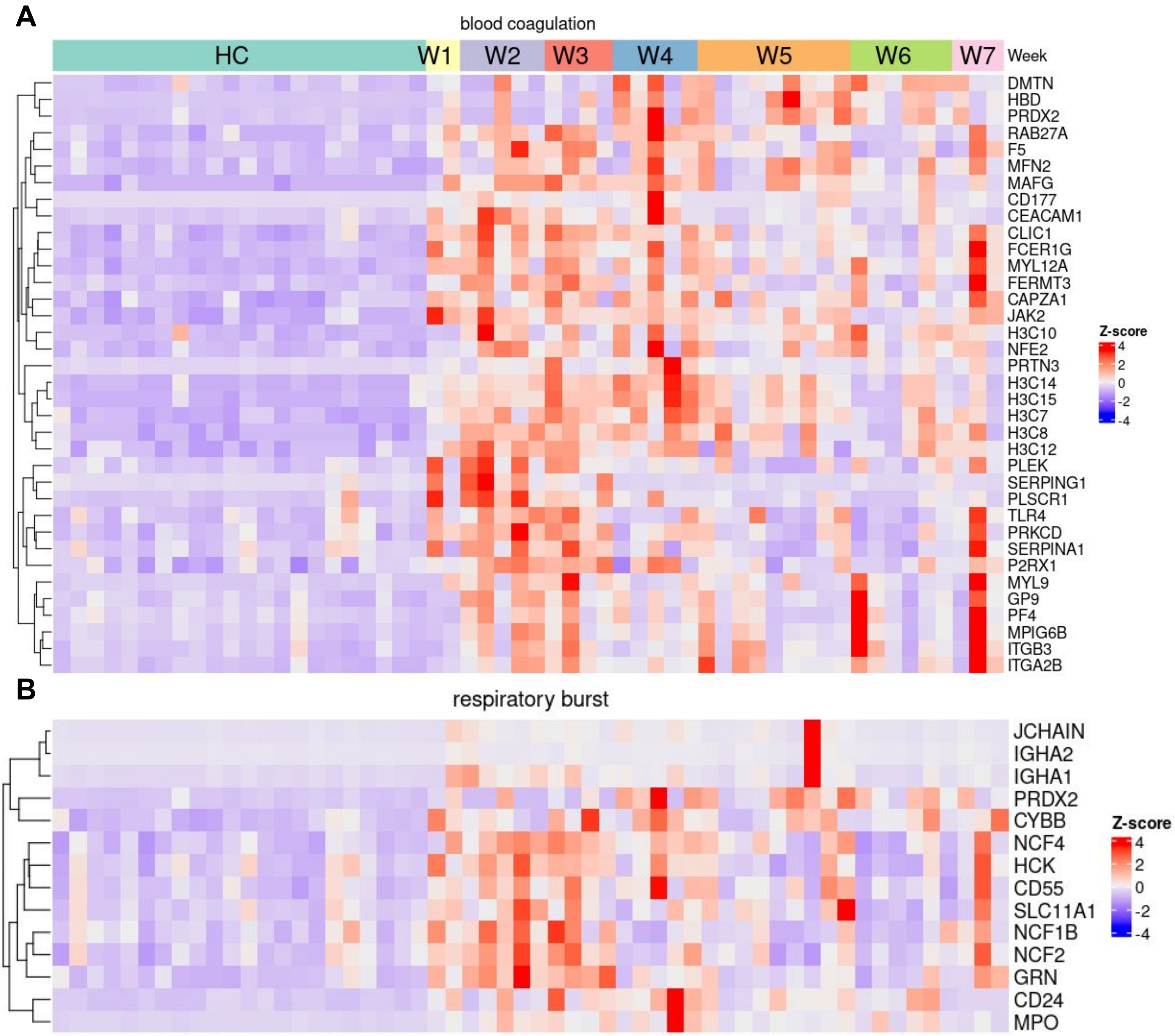

Supplementary Figure 8, related to Figure 4. Dynamic gene signatures of blood coagulation and respiratory burst pathways from severe COVID-19. (A) Severe COVID-19 DDEGs identified from the blood coagulation pathway. (B) Severe COVID-19 DDEGs identified from respiratory burst pathway. The heatmap is ordered by week following infection with healthy controls (HC) on the left. Color represents Z-score normalized expression values.

bioRxiv preprint doi: https://doi.org/10.1101/2021.05.04.442617; this version posted December 7, 2021. The copyright holder for this preprint (which was not certified by peer review) is the author/funder, who has granted bioRxiv a license to display the preprint in perpetuity. It is made available under aCC-BY 4.0 International license.

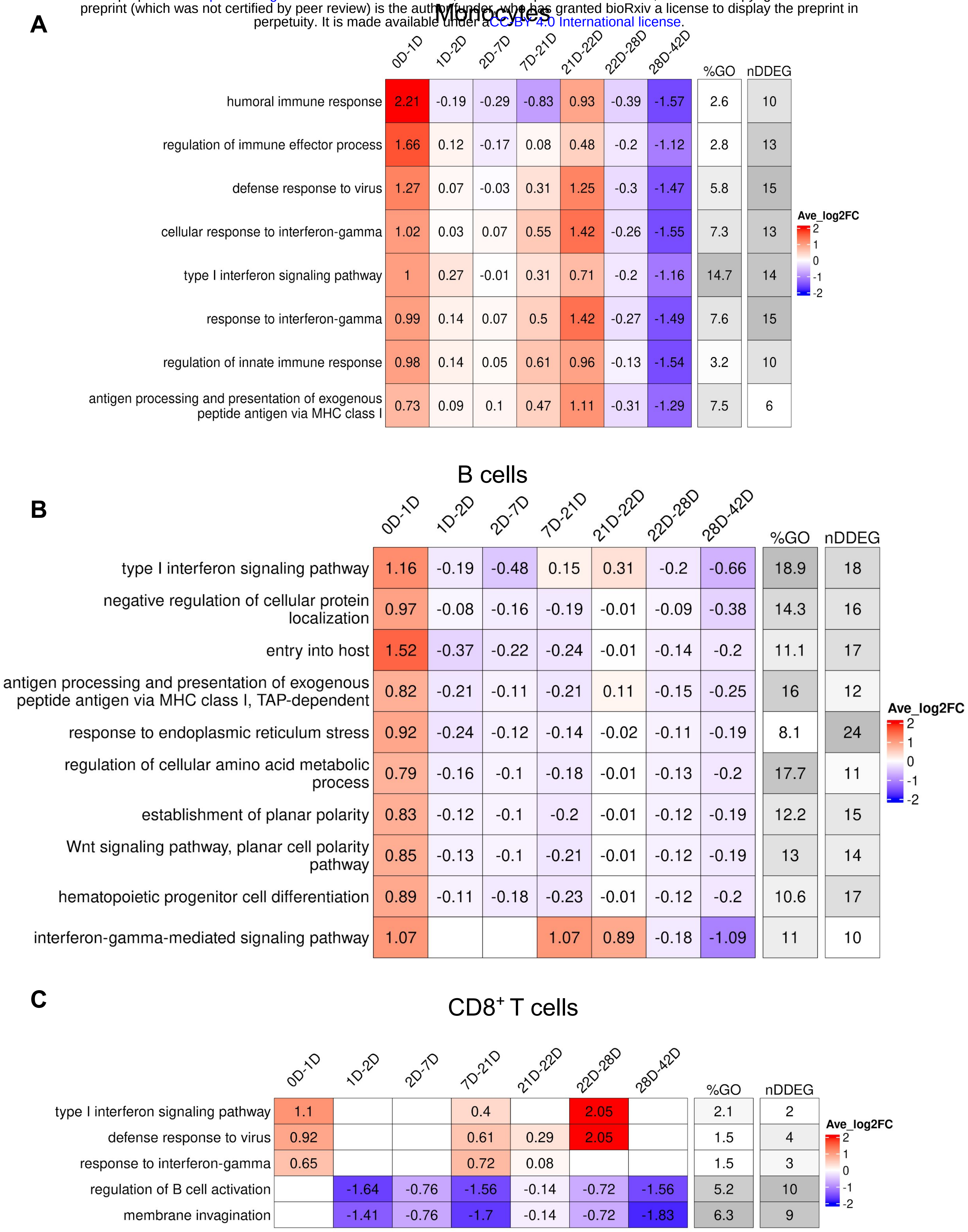

Supplementary Figure 9, related to Figure 6. TimeHeatmap of monocytes, B cells and CD8$^+$ T cells(A) *TimeHeatmap* of monocytes. (B) *TimeHeatmap* of B cells. (C) *TimeHeatmap* of CD8$^+$T cells. Day 1 is the first dose of vaccination, Day 21 is the second dose of vaccination.
